# Understanding climate change from a global analysis of city analogues

**DOI:** 10.1371/journal.pone.0217592

**Published:** 2019-07-10

**Authors:** Jean-Francois Bastin, Emily Clark, Thomas Elliott, Simon Hart, Johan van den Hoogen, Iris Hordijk, Haozhi Ma, Sabiha Majumder, Gabriele Manoli, Julia Maschler, Lidong Mo, Devin Routh, Kailiang Yu, Constantin M. Zohner, Thomas W. Crowther

**Affiliations:** 1 Crowther Lab, Department of Environmental Systems Science, Institute of Integrative Biology, ETH Zürich, Zürich, Switzerland; 2 Plant Ecology, Department of Environmental Systems Science, Institute of Integrative Biology, ETH Zürich, Zürich, Switzerland; 3 Department of Civil, Environmental and Geomatic Engineering, Institute of Environmental Engineering, ETH Zürich, Zürich, Switzerland; Universidade de Vigo, SPAIN

## Abstract

Combating climate change requires unified action across all sectors of society. However, this collective action is precluded by the ‘consensus gap’ between scientific knowledge and public opinion. Here, we test the extent to which the iconic cities around the world are likely to shift in response to climate change. By analyzing city pairs for 520 major cities of the world, we test if their climate in 2050 will resemble more closely to their own current climate conditions or to the current conditions of other cities in different bioclimatic regions. Even under an optimistic climate scenario (RCP 4.5), we found that 77% of future cities are very likely to experience a climate that is closer to that of another existing city than to its own current climate. In addition, 22% of cities will experience climate conditions that are not currently experienced by any existing major cities. As a general trend, we found that all the cities tend to shift towards the sub-tropics, with cities from the Northern hemisphere shifting to warmer conditions, on average ~1000 km south (velocity ~20 km.year^-1^), and cities from the tropics shifting to drier conditions. We notably predict that Madrid’s climate in 2050 will resemble Marrakech’s climate today, Stockholm will resemble Budapest, London to Barcelona, Moscow to Sofia, Seattle to San Francisco, Tokyo to Changsha. Our approach illustrates how complex climate data can be packaged to provide tangible information. The global assessment of city analogues can facilitate the understanding of climate change at a global level but also help land managers and city planners to visualize the climate futures of their respective cities, which can facilitate effective decision-making in response to on-going climate change.

## Introduction

The gap between the scientific and public understanding of climate change, referred to as the “Consensus Gap”, is largely attributed to failures in climate change communication[1]. Often limited to ad-hoc reporting of extreme weather events or intangible, long-term climate impacts (e.g. changes in average temperature by 2100). Despite an exhaustive list of risks associated to climate change [2] (e.g. heat stress, air and water quality, food supply, distribution of vectors of diseases, social factors), the intangible nature of reporting on climate change fails to adequately convey the urgency of this issue to a public audience on a consistent basis[3]. It is hard for most people to envision how an additional 2°C of warming might affect daily life. This ineffective communication of climate change facts, compounded by uncertainty about the extent of expected changes, has left the door open for widespread misinterpretation about the existence of this global phenomenon.

History has repeatedly shown us that data and facts alone do not inspire humans to change their beliefs or act [3]. Increased scientific literacy has no correlation with the acceptance of climate change facts [4]. A growing body of research demonstrates that visualization—the ability to create a mental image of the problem—is the most effective approach for motivating behavior change [5,6]. Several studies have analyzed ‘geographic shifts’ to better illustrate climate change. For example, Seidel and colleagues (2008) [7,8] showed that climate change has driven a widening of the tropical belt, by ~2 to 4.8 latitudinal degrees in recent decades. Similarly, the changing conditions of cities around the world provides another tangible example of shifting climate regimes. Given that over 50% of the global population exists within cities [9], these urban environments potentially valuable tool to visualize the impact of climate change at a global scale. As iconic locations, cities are associated with distinct sets of environmental conditions. As such, shifts in the climate conditions of these urban areas could provide a unique opportunity for people to visualize the impacts of climate change, and to establish effective response strategies to address the effects.

Several studies [10–15] and press reports [16,17] have shown that the use of ‘cities geographic shift’ or “city analogues” can help to understand and visualize the effects of climate change. In particular, cities can serve as useful climate analog, enabling people to visualize their own climate future via comparison with other cities that currently experience those climate conditions. However, until now, existing research have been focused on regional- or continent-scale analyses in North America or Europe [10–15], and we lack a unifying global perspective. These regional trends suggest that cities are likely to resemble those at lower latitudes as the climate continues to warm. However, it remains unclear if this trend holds at a global scale, as other climate drivers such as changing precipitation regimes may obscure these latitudinal trends. As such, Southern Hemisphere or tropical cities, which already exist in warm conditions and are likely to experience considerable changes in precipitation and extreme climate variation, may show independent geographic shifts under changing climate conditions. Generating a unified understanding of the shifts in the climate conditions of the world’s cities is critical if we are going to visualize the impacts of climate change in any biogeographic region. Generating this understanding requires a global perspective and the use of a full range of climate variables to represent the entire climate regime of those regions.

In this study, we evaluate the global shifts in the climate conditions of cities by taking current climate data for the world’s 520 major cities (Current Cities), and project what they will most closely resemble in 2050 (Future Cities). Rather than describing the quantitative changes in climate variables [18], we propose to quantify city climate analogs at a global scale [10–12], i.e. assessing which Current Cities will most closely resemble the climate conditions of Future Cities. To tackle previous limitations, we explore these patterns at a global scale using 19 bioclimatic variables, to include climate variability and seasonality in addition to climate averages.

Specifically, we aim to test three questions: (i) What proportion of the world’s major cities of the future most closely resemble their own current climate conditions *vs*. the climate conditions of other cities in different geographic regions? (ii) What proportion of cities will experience novel climate conditions that are outside the range experiences by cities today? (iii) If cities do shift their climate conditions, is this spatial shift uniform in direction across the planet?

## Materials and methods

### Selection of major cities

We selected these “major” cities of the world from the “LandScan (2016) High Resolution global Population Data Set” created by the Oak Ridge National Laboratory [19]. By “major” cities, we considered cities that are an administrative capital or that account more than 1,000,000 inhabitants. In total, 520 cities were selected.

### The climate database

To characterize the current climate conditions among these major cities of the world, we extracted 19 bioclimatic variables from the latest Worldclim global raster layers (Version 2; period 1970–2000) at 30 arc-seconds resolution [20]. These variables captured various climatic conditions, including yearly averages, seasonality metrics, and monthly extremes for both precipitation and temperature at every location.

### Future data: GCMs, downscaling and future scenarios

For the future projections, the same 19 bioclimatic variables were averaged from the outputs of three general circulation models (GCM) commonly used in ecology [21,22]. Two Community Earth System Models (CESMs) were chosen as they investigate a diverse set of earth-system interactions: the CESM1 BGC (a coupled carbon–climate model accounting for carbon feedback from the land) and the CESM1 CAM5 (a community atmosphere model) [21]. Additionally, the Earth System component of the Met Office Hadley Centre HadGEM2 model family was used as the third and final model [22]. To generate the data, we chose Representative Common Pathway 4.5 (RCP 4.5) scenario from the Coupled Model Intercomparison Project Phase 5 (CMIP5) as the input. It is a stabilization scenario, meaning that it accounts for a stabilization of radiative forcing before 2100, anticipating the development of new technologies and strategies for reducing greenhouse gas emissions [23]. By using this optimistic climate change scenario, we represent conservative changes in climate conditions that are likely to occur even if substantial climate change mitigation occurs. For each output, a delta downscaling method developed by the CGIAR Research Program on Climate Change, Agriculture and Food Security (CCAFS) was applied to reach a resolution of 30 arc-seconds [24], using current conditions Worldclim 1.4 as a reference. Downscaling approach were necessary to assess climate conditions at the cities’ scale even if it induces a risk of pixel mismatch and consequently, a lower level of confidence for local scale analyses [25,26].

### Summarizing the current climate among the major cities through a principal component analysis

The 19 current and future bioclimatic variables were extracted from the coordinates of the 520 major cities (i.e., the city centroids), meaning each city had two sets of bioclimatic metrics: the current climate data for the world’s major cities (Current Cities) and the equivalent 2050 projection (Future Cities) according to the average of the three RCP 4.5 GCMs.

**Fig 1:**
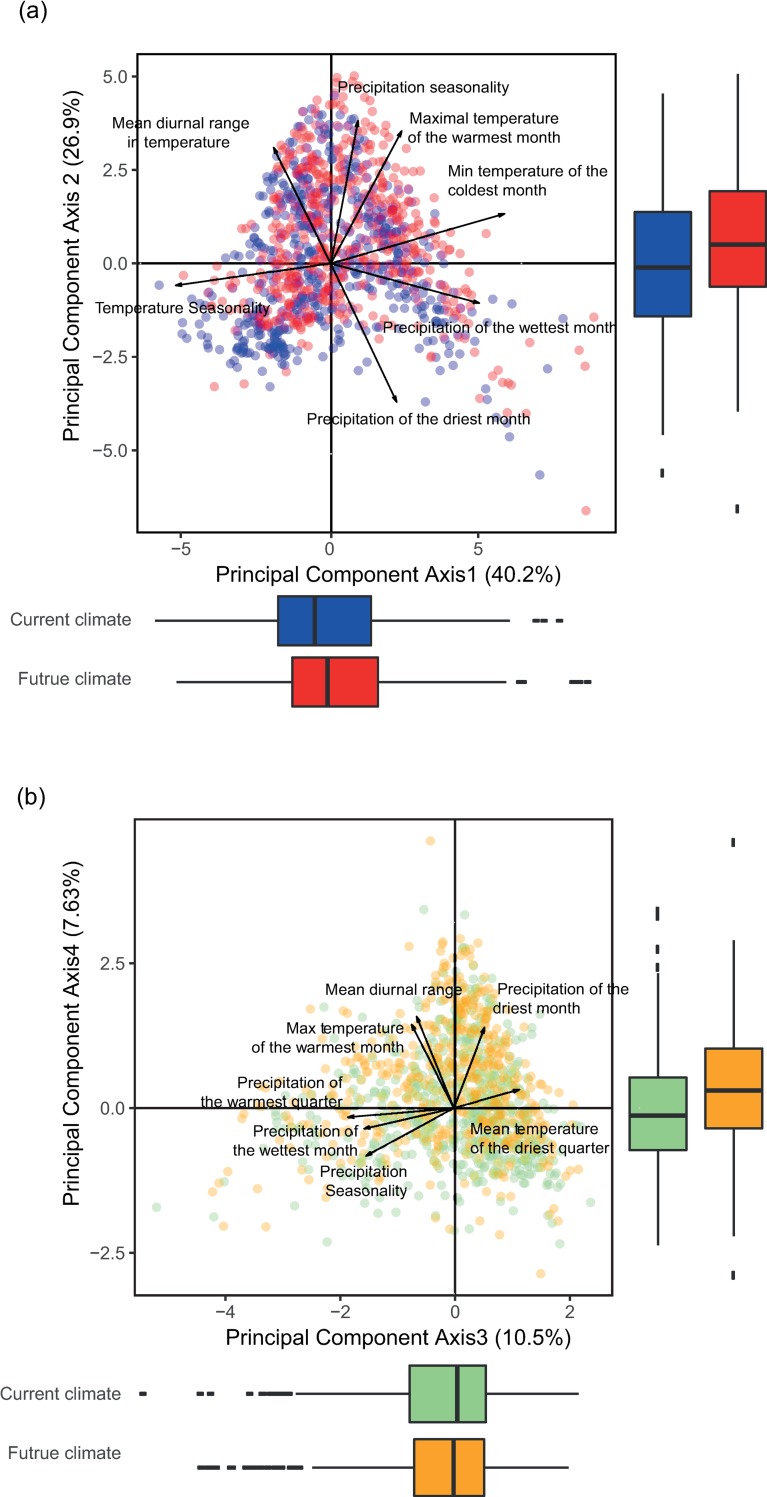
Distribution of current and future cities along the first 4 principal component axes. The seven major climate variables contributing to the Principal Component Analysis (PCA) are superposed on each figure. The figure at the top (a) shows the distribution of current (blue) and future (red) cities on the space defined by the first two principal components. The first two axes explain, respectively, 40.2 and 26.9% of climate variations. The first axis is mainly driven by differences in temperature seasonality and in minimum temperature of the coldest month, while the second axis is mainly driven by differences in precipitation seasonality. The figure at the bottom (b) shows the same current (green) and future (orange) cities on the space defined by the third and fourth principal components. They explain respectively 10.5 and 7.6% of climate variations. The third axis is mainly driven by changes in precipitation of the wet season, while the fourth axis is mainly driven by changes in the mean diurnal temperature range. Boxplots illustrates the distribution of the points along each of the 4 axes. The continuous line in the boxes represents the median of the distribution, the extremities of the boxes the 1^st^ and the 3^rd^ quartile and the continuous lines go up to 1.5 times the difference between the 3^st^ and the 1^rd^ quartile.

A scaled principal components analysis (PCA) was performed on current bioclimatic data in order to account for correlation between climate variables and to standardize their contributions to the subsequent dissimilarity analysis [27]. As the first four principal components accounted for more than 85% of the total variation of climate data (40.2%, 26.9%, 10.5% and 7.6%, respectively), the remaining principal components were dropped from later analyses. The main contributing variables to the four components are the temperature seasonality (axis 1), the minimum temperature of the coldest month (axis 1), the maximum temperature of the warmest month (axis 2), the precipitation seasonality (axis 2), the precipitation of the driest (axis 4) and of the wettest (axis 3) month, and the temperature diurnal range (axis 4, Fig 1).

### Calculating the extent of the covered climate domain

For further interpretation of the results, a convex hull was computed from the coordinates of the Current Cities within the multivariate space defined by the first four principal components axes [28]. For reference, a convex hull of a set of N-dimensional points forms the smallest possible hypervolume (in N-dimensions) containing all points defined in that set; in this case, it defines the bounds of climatic combinations that Earth currently experiences in these 520 cities. All Future Cities falling outside the hypervolume of this convex hull represent currently non-existent bioclimatic assemblies in these cities, i.e. cities with no current climate analog [29].

### Pairing cities based on the similarity between current and future climate conditions

Euclidean distances (i.e., dissimilarity indices) were calculated for every combination of Current and Future City based on their coordinates within the multivariate space defined by the first four principal components axes, creating a symmetric dissimilarity matrix with pairwise comparisons for all cities (S1 Table). The Euclidean distance was calculated using the vegan package on R (RCran version 3.3.2) [30]. Each Future City was then paired with its three closest Current Cities based on the dissimilarity values (S1 Table, S2 Table). Three cities are kept for each Future city in order to facilitate comparison between Current and Future climate, as all cities are not necessarily known by the reader. To avoid un-realistic shifts or shifts due to pixel mismatch between Current and Future climate conditions, the final analysis was performed keeping shift values between the 5^th^ and the 95^th^ percentile, i.e. keeping 477 out of the original 520 cities.

### Calculating the absolute latitudinal shift

To illustrate and summarize the shifts between Current and Future Cities, we calculated the importance of absolute latitudinal shift for each city. Shifts in latitude were standardized for both hemisphere, so that a shift south in the northern hemisphere is equal to a shift north in the southern hemisphere, i.e. referred as the absolute latitudinal shift. In other words, the absolute latitudinal shift expresses a geographic shift in relation to the equatorial line (shifting away from or towards the equator).

Analyses and figures were performed using R, maps were built using Q-GIS 3.0.

## Results

### Analysis of changes between current and future cities from the PCA

The future climate of each city was projected within the four principal components (using the PCA eigenvectors derived from the bioclimatic variables of the current climate) to allow for direct comparison between Current and Future Cities (Fig 1). On the plane defined by the first two components of the PCA (Fig 1A), explaining respectively 40.2 and 26.9% of climate variations, we observe changes towards less temperature seasonality, with higher maximal and minimal temperatures during the year, as well as higher precipitation seasonality, with higher precipitation in the wettest month but lower precipitation in the driest one. While no clear trend can be observed along the third axis (10.5% of climate variation), the changes along the fourth axis (7.6% of climate variation) show higher temperature diurnal range (Fig 1B), i.e. the daily difference between cities’ maximum and minimum temperatures will increase. In brief, cities of the world become hotter, in particular during the winter and the summer. Wet seasons become wetter and dry season drier.

### What proportion of cities will resemble their own current climate *vs*. other cities by 2050?

We characterized the climate of the world’s 520 major cities using 19 climatic variables that reflect the variability in temperature and precipitation regimes for current and future conditions. Future conditions are estimated using an optimistic Representative Concentration Pathway (RCP4.5), which considers a stabilization of CO_2_ emissions by mid-century (see Material and Methods). This model was chosen to show the extent of the changes we would be facing even considering the implementation of effective mitigation policies. Using a multivariate analysis, we analyzed the climate similarity of all Current and Future cities to one another (S1 Table). This simple analysis enables us to estimate which major cities of the world will remain relatively similar, and which will shift to reflect the climate of another city by 2050. Overall, our analysis shows that 77% of the world’s Current Cities will experience a striking change in climate conditions, making them more similar to the conditions of another existing city than they are to their own current climate conditions (S1 Table, S2 Table). The climate conditions of remaining 23% of cities remained most closely associated with their current climate conditions.

### What proportion of cities will experience novel climate conditions?

**Fig 2:**
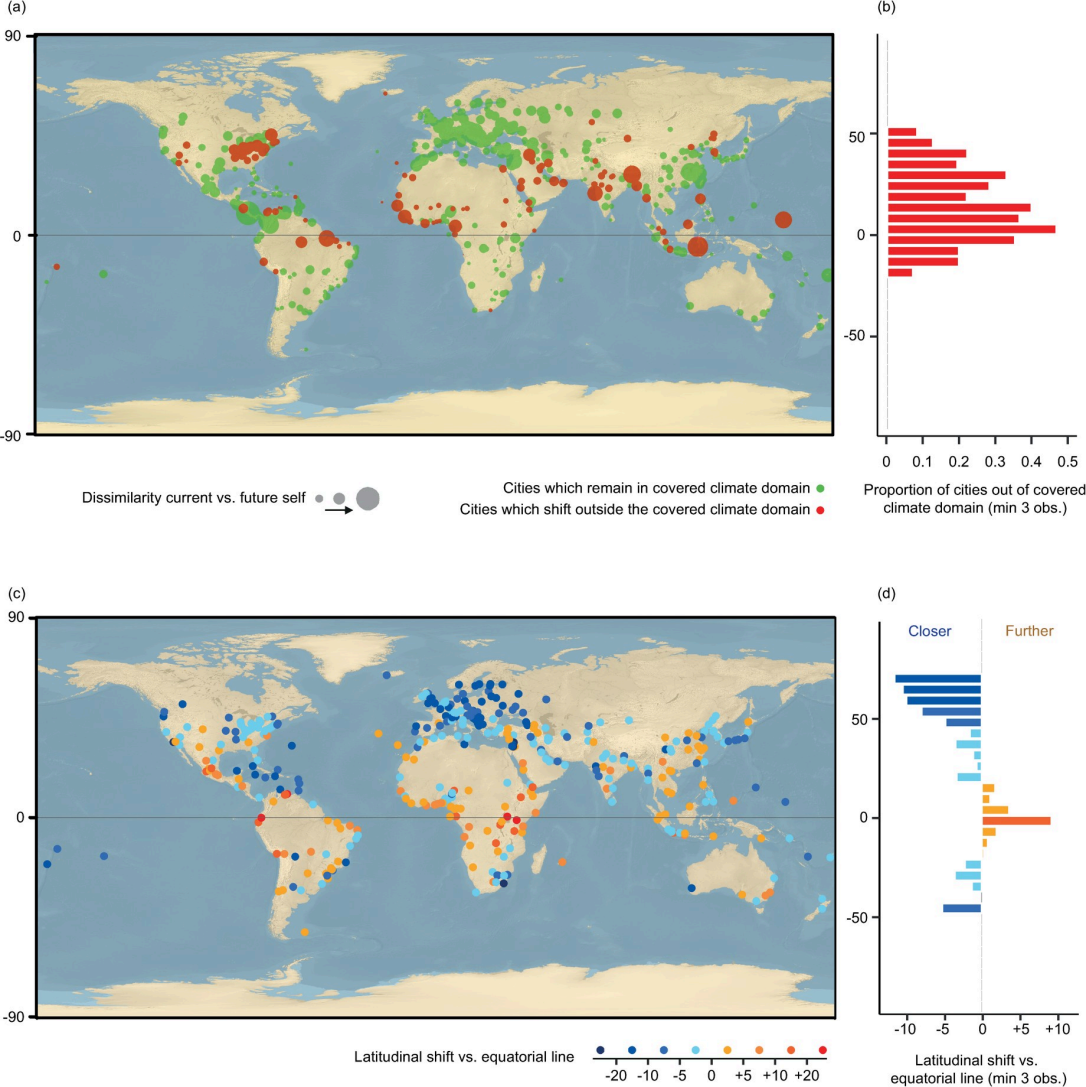
Extent of climate changes in major cities of the world by 2050. **a, b,** the extent of change in climate conditions. Cities predicted to have climates that no major city has experienced before are colored in red (mostly within the tropics). Cities for which future climate conditions reflect current conditions in other major cities of the world are shown in green. The size of the dots represents the magnitude of change between current and future climate conditions. **b**, The proportion of cities shifting away from the covered climate domain (concentrated in the tropics). **c,d,** The extent of latitudinal shifts in relation to the equatorial line. Cities shifting towards the equator are colored with a blue gradient (mostly outside the tropics), while cities shifting away from the equator are colored with a yellow to red gradient (mostly within the tropics). **d,** A summary of the shift by latitude is illustrated in a barchart, with shifts averaged by bins of 5 degrees. The background of the maps are a combination rasters available in the public domain, i.e. of USGS shaded relief only and hydro cached.

Overall 78% of the 520 Future Cities studied present a climate within the hypervolume representing covered combinations of climate conditions. Therefore, 22% of the Future Cities’ climate conditions would disappear from this current climatic domain (Fig 2A). As such, 22% of the world’s cities are likely to exist in a climatic regime that does current exist on the planet today. The situation is even more pronounced in the tropics, with 30% of cities experiencing novel climate conditions essentially because the climate will get drier.

### Is this spatial shift uniform in direction across the planet?

**Fig 3:**
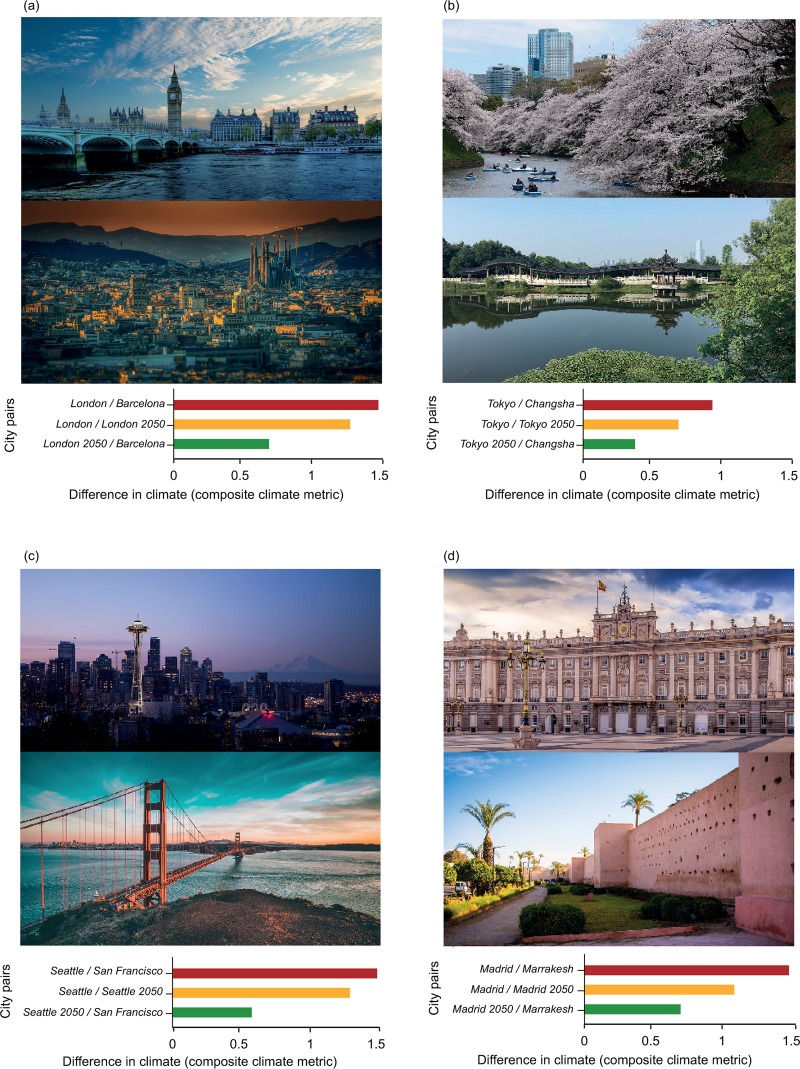
Future cities and similar current climate counterpart. Difference between future and current climate for four cities and an example of their similar current counterpart. Illustration of the results of the analysis for London (**a**; counterpart: Barcelona), Buenos Aires (**b**; counterpart: Sidney), Nairobi (**c**; counterpart:Beirut) and Portland (**d**; counterpart:San Antonio). The red bar represents the difference between the current climate of the city of interest (e.g. London in (a)) and the current climate of the city to which the city of interest (e.g. London in (a)) will have the most similar climate by 2050 (e.g. Barcelona in (a)). The yellow bar the difference between the current and future climate of the city of interest (e.g. current London and London 2050 in (a)). The green bar represents the difference between the future climate of the city of interest (London 2050) and the current climate of the most similar counterpart (e.g. Barcelona in (a)). Images of Barcelona and London were obtained on Pixabay, shared under common creative CC0 license.

The proportion of shifting cities varied consistently across the world. Cities in northern latitudes will experience the most dramatic shifts in extreme temperature conditions (Fig 2C and Fig 2D). For example, across Europe, both summers and winters will get warmer, with average increases of 3.5°C and 4.7°C, respectively. These changes would be equivalent to a city shifting ~1,000 km further south towards the subtropics, i.e. a velocity ~20 km.year^-1^, under current climate conditions (Fig 2C and Fig 2D). Consequently, by 2050, striking changes will be observed across the northern hemisphere: Madrid’s climate in 2050 will be more similar to the current climate in Marrakech than to Madrid’s climate today; London will be more similar to Barcelona, Stockholm to Budapest; Moscow to Sofia; Portland to San Antonio, San Francisco to Lisbon, Tokyo to Changsha, etc(Fig 3, S2 Table).

Cities in the tropical regions will experience smaller changes in average temperature, relative to the higher latitudes. However, shifts in rainfall regimes will dominate the tropical cities. This is characterized by both increases in extreme precipitation events (+5% rainfall wettest month) and, the severity and intensity of droughts (-14% rainfall driest month). With more severe droughts, tropical cities will move towards the subtropics, i.e. towards drier climates (Fig 2C and Fig 2D). However, the fate of major tropical cities remains highly uncertain because many tropical regions will experience unprecedented climate conditions. Specifically, of all 22% of cities that will experience novel climate conditions, most (64%) are located in the tropics. These include Manaus, Libreville, Kuala Lumpur, Jakarta, Rangoon, and Singapore (Fig 2A and Fig 2B, S2 Table).

**Fig 4:**
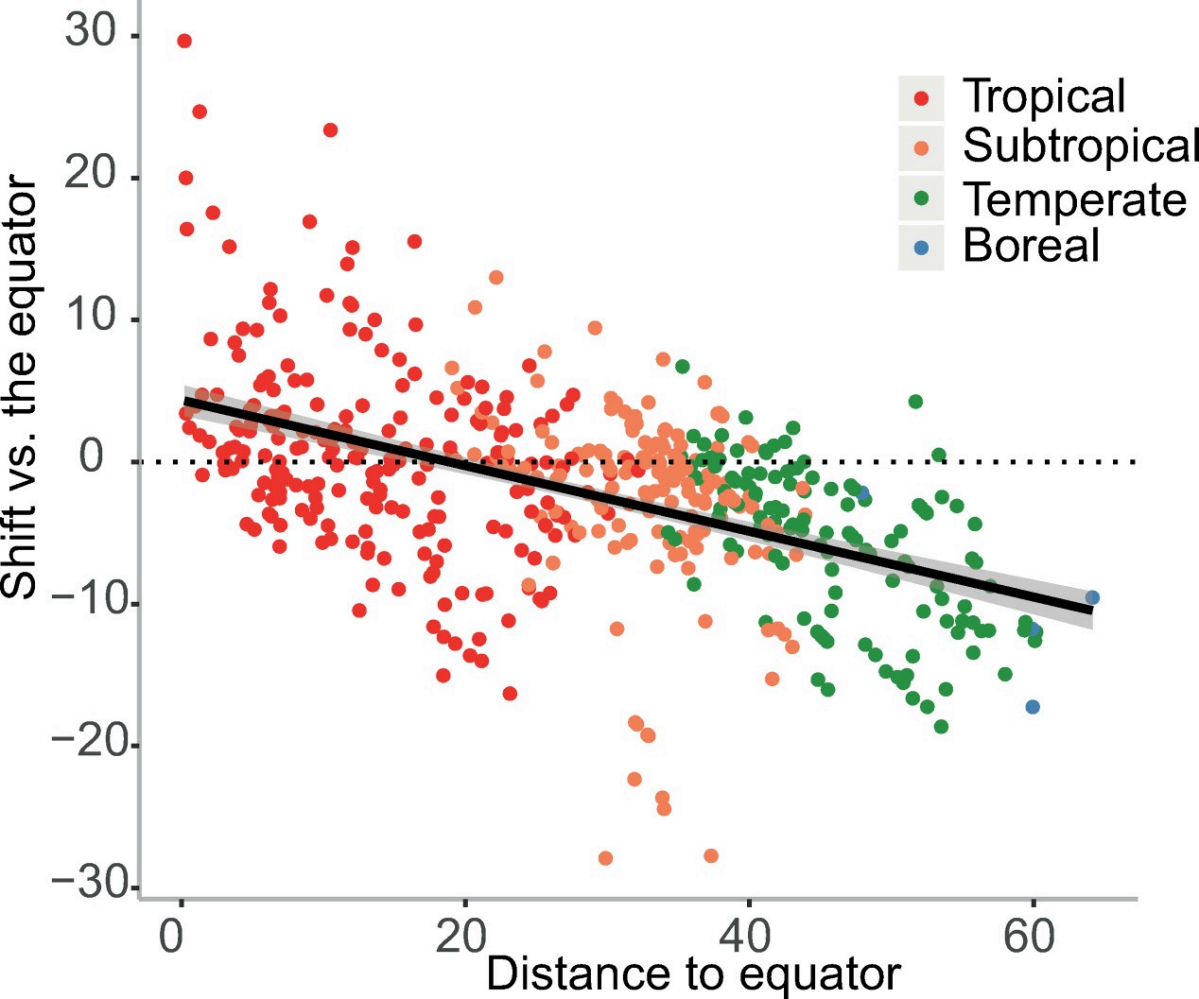
Latitudinal shift of cities relative to their distance to the equator (in degrees). Cities below 20 degrees North/South tend to move away from the equator (positive latitudinal shift) while cities beyond 20 degrees North/South tend to move closer to the equator (negative latitudinal shift). Cities are colored according to the aggregated ecoregion of the world [36] to which they belong, with the tropical in red, the subtropical in orange, the temperate in green and the boreal in blue.

In summary, at a global level, we observe a global geographic shift towards the subtropics, i.e. towards ~20 degrees of latitude (Fig 2B and Fig 4).

## Discussion

Our analysis reveals consistent global patterns in the climate shifts of future major cities around the world over the next 30 years. Despite our use of a highly optimistic climate change scenario (i.e. RCP 4.5), we show that the climate conditions of over 77% of world’s major cities will change to such a great extent that they will resemble more closely the conditions of another major city. The projected shifts showed consistent biogeographic trends, with all city climates (both southern and northern hemisphere) generally shifting towards the conditions in warmer, low-latitude regions. The extent and consistency of these patterns provides a stark reminder of the global scale of this climate change threat and associated risks for human health. In contrast to previous analyses, our analysis also reveals that 22% of the world’s cities are likely to exist in a climatic regime that does not current exist on the planet today. These trends highlight the extreme vulnerability of tropical and sub-tropical cities, 30% of which will experience shifts into entirely novel climate regimes with no existing analogues across the world’s major cities. This lends support to the idea of novel climates, which are expected to emerge in many tropical and sub-tropical regions [29]. It should be noted that, by defining the climate envelope using a convex-hull (i.e. by defining a volume from simplices (“triangles”) that form the smallest convex simplicial complex of a set of input points in 4-dimensional space), we applied a conservative method for evaluating future change. Indeed, because it includes the smallest level of extrapolation and generating the smallest possible shapes, this approach has a low-risk of incorrectly identifying novel climate conditions, relative to a concave-hull approach [31]. However, this approach necessarily comes with the high likelihood of missing some novel climates. The 22% of cities experiencing a novel climate must therefore be seen as a highly conservative estimate.

Our findings also support previous studies conducted in Europe [10,11] and north America [13], stressing the current trend of north-to-south geographical shift across the northern hemisphere. Yet, using an optimistic climate change scenario, we found that the velocity (i.e. the speed of geographical shift) risks to be higher in the near future than in the second half of the 21th century [10] passing from 15 km year^-1^ to 20 km year^-1^. Our study also allows the extension of such observations to the global scale, showing that observations for Europe can be generalized for the entire Northern Hemisphere and for a part of the southern hemisphere (Fig 2B). At the global scale, our study reveals that geographical shift tend to converge towards the subtropics (Fig 4), going to warmer climate conditions from boreal and temperate regions and to drier conditions from tropical regions. While this lends support to previous observations of a “tropical belt widening” due to the expected warmer conditions [7,8], it also shows that tropical biomes tend to shrink in many areas due to drier conditions. We therefore suggest here to refer to a “sub-tropical widening” compared to the previous “tropical widening” due to climate change.

While our findings are necessarily dependent on the methodology used to identify the climatic shifts, it is widely recognized that the choice of the metric to assess the similarity-dissimilarity of the climate conditions between cities has an extremely minor effect, compared to the choice of the climate model and scenario[32]. That is, our results are unlikely to be affected whatever method we use to calculate dissimilarity, as the variation between climate projections is far greater. Nonetheless, Mahony and colleagues [31] highlighted the need to standardize the contribution of each climate variable to the dissimilarity matrix and to account for correlation between them to avoid any bias[31]. In the present study, we address this using a scaled principal component analysis to summarize the main bioclimatic variations among the 520 major cities. This approach simply follows classic dissimilarity analysis recommendations for ecological studies[27], applying an Euclidean distance matrix on the main dimensions of the principal component analysis to assess the similarity between cities. This method was preferred to the sigma-dissimilarity developed by Mahony and colleagues[31] for its simplicity and it broad use in ecological sciences.

Our analysis allows us to visualize a tangible climate future of the world’s major cities. These results enable decision makers from all sectors of society, to envision changes that are likely to occur in their own city, within their own lifetime. Londoners, for example, can start to consider how their 2050 equivalents (e.g. Barcelona today) have taken action to combat their own environmental challenges. In 2008, Barcelona experienced extreme drought conditions, which required the importation of €22m of drinking water. Since then, the municipal government has implemented a series of ‘smart initiatives‘ to manage the city’s water resources (including the control of park irrigation and water fountain levels). The Mayor of London has factored drought considerations into his Environment Strategy aims for 2050 [33], but this study can provide the context to facilitate the development of more targeted climate strategies. In addition, this information can also empower local citizens to evaluate proposed environmental policies. By allowing people to visualize their own climate futures, we hope that this information can facilitate efforts to mitigate and adapt to climate change.

Our study is not a novel model revealing updated climate projections or expectations by 2050. Instead, our analysis is intended to illustrate how complex climate data can be effectively summarized into tangible information that can be easily interpreted by anyone. Of course, the climate scenarios that we have used are based on predictions from a few climate models, run under a single (business as usual) climate scenario. We recognize that these models are characterized by huge amounts of uncertainty [34], and the predicted Future Cities may change as these Earth System Models are refined, in particular in light of urban climate specificities [35]. However, our results are likely to reflect the qualitative direction of climate changes within cities and so meet our primary goal, which is to communicate predicted climate changes to a non-specialist audience in order to motivate action. When model projections are updated, we would recommend communicating any new results with this goal in mind.

### Conclusion

To our knowledge, our study represents the first global analysis of the shifts in climate conditions of the world’s major cities under climate change. Our analysis revealed that over 77% of the world’s cities are likely to experience a shift towards the climate conditions of another major city by 2050, while 22% will shift to climate conditions that are not currently present for any major cities on the planet. Across the globe, the direction of movement is generally trending towards the subtropics, providing unifying patterns that support trends observed in Europe and North America. In addition, this analysis revealed new insights for cities in equatorial regions, many of which are likely to move to entirely new climate conditions that are not currently experienced by any of the other global cities today. These city analogues, and the data we openly share, can help land managers and city planners to visualize the climate futures of their respective cities, facilitating efforts to establish targeted climate response strategies. As well as facilitating our basic understanding of climate change effects, our analysis highlights the value of using cities to visualize the tangible effects of climate change across the globe.

## Supporting information

S1 Table Dissimilarity between current and future climate of the major cities of the world. The dissimilarity is expressed as the Euclidean distance matrix performed on the 4 main axes of the PCA analysis that summarizes the climate variation (19 bioclimatic variables) among the major cities of the world.
(ODS)

S2 Table Summary statistics of the global analysis of city analogues. The table provides the three cities for which current climate is the most similar to the future climate of each city. It also provides the associated latitudinal shift for the most similar city and the expected changes in climate conditions by 2050 for the mean annual temperature, the annual precipitations, the temperature of the warmest month, the temperature of the coldest month and the precipitation of the wettest month.
(ODS)
