# Correction: Understanding climate change from a global analysis of city analogues

**DOI:** 10.1371/journal.pone.0224120

**Published:** 2019-10-16

**Authors:** Jean-Francois Bastin, Emily Clark, Thomas Elliott, Simon Hart, Johan van den Hoogen, Iris Hordijk, Haozhi Ma, Sabiha Majumder, Gabriele Manoli, Julia Maschler, Lidong Mo, Devin Routh, Kailiang Yu, Constantin M. Zohner, Thomas W. Crowther

There is an error in the third sentence of the “What proportion of cities will experience novel climate conditions?” subsection of the Results. The correct sentence is: As such, 22% of the world’s cities are likely to exist in a climatic regime that does not currently exist on the planet today.

There is an error in reference 30. The correct reference is: Cormack RM, Legendre L, Legendre P. Numerical Ecology. Biometrics. Elsevier; 2006;40: 280.

There is an error in reference 33. The correct reference is: London M of. London Environment Strategy. 2018; https://www.london.gov.uk/sites/default/files/london_environment_strategy.pdf.

The Data Availability statement for this paper is incorrect. All relevant data are not provided in the published paper and its Supporting Information files. The authors have provided the data as Supporting Information S1–S3 Files. Additionally, the authors have provided guidelines for replicating the results of their paper. The guidelines are as follows:

1. Compute the 19 bioclimatic variables from the present (WORLDCLIM2) and future for the data specifically mentioned in the Methods section. The transformation, corrections, etc. applied on these are detailed on the CCAFS-CLIMATE website (referenced below). The authors used the downscaled model developed from CGIAR-CCAFS, scenario RCP4.5, for 2050. Following these instructions leads here: http://ccafs-climate.org/file-list.php?tile_name=&scenarios%5B%5D=8&extent=1&period%5B%5D=6&variables%5B%5D=1&resolution=1.
2. Build the City-Climate tables, one for the present, one for the future.
3. Compute a PCA of the 19 bioclimatic variables for the 1970–2000 to standardize the contribution of each variable and avoid autocorrelation issues (avoid double accounting of the same information).
4. Keep the 4 main axes.
5. Project the 19 bioclimatic variables from 2050 in these 4 axes, 4 axes built from the 1970–2000 conditions.
6. Perform a distance matrix between the two tables (each table has 4 values for each city, related to the 4 axes of the PCA summarizing the 19 bioclimatic variables).
7. Assess which cities are similar to each other.

## Supporting information

S1 File (R)

S2 File (CSV)

S3 File (CSV)
